# Lessons From a Crisis: A Thematic Analysis on Occupational Stress in Staff in an Acute Paediatric Teaching Hospital in Ireland

**DOI:** 10.1192/bjo.2024.270

**Published:** 2024-08-01

**Authors:** Shay Ward, Grainne Donohue, Fiona McNicholas, Johanna Murray

**Affiliations:** 1 Saint John of God Community Mental Health Services, Dublin, Ireland; 2 Saint Patrick's University Hospital, Dublin, Ireland; 3 Children's Health Ireland Crumlin Hospital, Dublin, Ireland

## Abstract

**Aims:**

The COVID-19 pandemic placed increased pressure on service provision and healthcare worker [HCW] wellness. As the pandemic recedes, staff need an appropriate response to facilitate individual and organisational recovery, to minimise long-term healthcare worker burnout and to be better equipped for future crisis in healthcare. The aim was to explore and reflect on the experiences of staff working during the COVID-19 pandemic in an acute paediatric hospital to determine an appropriate response in the post-crisis work environment.

**Methods:**

A Qualitative research design using responses from open ended questions from one hundred and thirty-three clinical and non-clinical staff (89% clinical) from an Irish paediatric teaching hospital. Reponses were thematically analysed.

**Results:**

HCWs experienced frustration, uncertainty, anxiety and stress, during the pandemic crisis. This included communication inconsistencies, inadequate support and staffing and other resource shortages, leaving staff at high risk for long-term burnout as the pandemic recedes. Three themes were developed detailing this; 1) Support, 2) Communication and 3) Trust.

**Conclusion:**

This research supports the long-standing need to increase mental health service investment and to implement an appropriate response to regain and maintain a healthy workforce, post COVID-19. This response should address the biopsychosocial needs of the individual and healthcare organisations should work dynamically, creatively and collaboratively to ensure the psychological safety of its workforce moving forward.

